# Modified motor unit properties in residual muscle following transtibial amputation

**DOI:** 10.1088/1741-2552/ad1ac2

**Published:** 2024-01-17

**Authors:** Noah Rubin, Robert Hinson, Katherine Saul, William Filer, Xiaogang Hu, He (Helen) Huang

**Affiliations:** 1 UNC/NC State Joint Department of Biomedical Engineering, North Carolina State University, Raleigh, NC 27695, United States of America; 2 UNC/NC State Joint Department of Biomedical Engineering, University of North Carolina at Chapel Hill, Chapel Hill, NC 27599, United States of America; 3 School of Medicine, University of North Carolina at Chapel Hill, Chapel Hill, NC 27599, United States of America; 4 Department of Mechanical & Aerospace Engineering, North Carolina State University, Raleigh, NC 27695, United States of America; 5 Department of Mechanical Engineering, Pennsylvania State University, University Park, PA 16802, United States of America

**Keywords:** amputation, firing rate, motor unit, recruitment threshold, surface electromyogram

## Abstract

**Objective.:**

Neural signals in residual muscles of amputated limbs are frequently decoded to control powered prostheses. Yet myoelectric controllers assume muscle activities of residual muscles are similar to that of intact muscles. This study sought to understand potential changes to motor unit (MU) properties after limb amputation.

**Approach.:**

Six people with unilateral transtibial amputation were recruited. Surface electromyogram (EMG) of residual and intact *tibialis anterior* (TA) and *gastrocnemius* (GA) muscles were recorded while subjects traced profiles targeting up to 20% and 35% of maximum activation for each muscle (isometric for intact limbs). EMG was decomposed into groups of MU spike trains. MU recruitment thresholds, action potential amplitudes (MU size), and firing rates were correlated to model Henneman’s size principle, the onion-skin phenomenon, and rate-size associations. Organization (correlation) and modulation (rates of change) of relations were compared between intact and residual muscles.

**Main results.:**

The residual TA exhibited significantly lower correlation and flatter slopes in the size principle and onion-skin, and each outcome covaried between the MU relations. The residual GA was unaffected for most subjects. Subjects trained prior with myoelectric prostheses had minimally affected slopes in the TA. Rate-size association correlations were preserved, but both residual muscles exhibited flatter decay rates.

**Significance.:**

We showed peripheral neuromuscular damage also leads to spinal-level functional reorganizations. Our findings suggest models of MU recruitment and discharge patterns for residual muscle EMG generation need reparameterization to account for disturbances observed. In the future, tracking MU pool adaptations may also provide a biomarker of neuromuscular control to aid training with myoelectric prostheses.

## 1. Introduction

In recent decades a wide body of research has investigated the use of neural signals of residual muscles to control both upper- [1] and lower-limb [2] powered prostheses in people after amputation. By directly integrating volitional motor intent of a user into the control loop, myoelectric control of prosthesis dynamics provides an intuitive control scheme, with promise to restore functionality of a lost joint. Among recording techniques, surface electromyography (EMG), a noninvasive measure of neural signals amplified during muscle activity, is frequently implemented to decode user motor intent and drive myoelectric prosthesis control.

Current model-based myoelectric controllers [3–6] that relate neural activation of muscle to joint dynamics account by mapping EMG signals to motor output assume activation of residual muscles is similar to that of intact muscles, despite marked alterations following amputation [7, 8]. For example, in amputation, partial excision and detachment of muscles and tendons occurs, and damage to the peripheral nervous system disrupts afferent feedback [9]. Because native biomechanical function of residual muscles is lost, internal representations of central motor control also become modified [10]. With these severe disturbances to the neural, muscular, and skeletal systems, it raises the question whether assumptions regarding neural activation of residual muscle and the corresponding EMG signals are valid. Therefore, the goal of the current study was to understand mechanisms of neural activation of residual muscles. Specifically, we intended to identify possible changes in motor unit (MU) recruitment and discharge patterns associated with the peripheral injury of amputation.

Limited research aimed at understanding how limb amputation affects neuromuscular control of residual muscles in either upper- or lower-limbs has been reported. Little work has studied changes in neuromuscular control in the upper-limb, potentially because the structure of upper-limbs brings higher complexity to study with a small region controlling many degrees of freedom. Focusing on the lower-limb, earlier studies have measured performance of users when targeting specific levels of muscle activation ballistically between two residual agonist-antagonist ankle muscles in people with transtibial amputation [11]. Building off this work, a similar approach explored patterns of control in reciprocal coactivation of residual ankle muscles [12] and extended the paradigm to observe control performance for postural stability [13]. Others have investigated symmetry of muscle activation between lower-limbs in bilateral tasks [14]. Among these studies, most analyses on residual neuromuscular control have been limited to macro-level features of the EMG signal (such as amplitude and activation timing), ignoring complex signal content that may reveal additional alterations following amputation. The signal is generated from the summation of MU action potentials (MUAPs) [15], where MUs are motor neurons and all respectively innervated muscle fibers. Thus, EMG amplitude only indirectly relates to neural input to muscles, and this feature is biased to activity of motor neurons that innervate a larger number of muscle fibers [16, 17]. Besides a preliminary study suggesting MU firing rates are lower with higher variability in residual muscle compared to MUs in intact muscle [7], little investigation on neuromuscular control of residual muscle at the MU-level has been conducted.

MUs are widely studied, as they are the smallest functional unit of neuromuscular control, bridging inputs from the central nervous system to outputs of muscle activation [18]. To examine neuromuscular control, for decades single MU activities have been analyzed through intramuscular electrodes [19], which significantly advanced our understanding of MU recruitment [20] and firing patterns [21]. More recently, electrode designs have advanced in parallel with EMG decomposition algorithms, allowing groups of MUs generating EMG to be isolated [22]. This technique has permitted analysis on neural coding for muscle activation separate from anatomical properties of the muscle [16, 17], and noninvasive observation of MU physiology at the populational level [23]. Collectively, many studies suggest MUs tend to be recruited and fire in systematic patterns. These patterns have been modeled as functions relating MU physiology and implemented in well-established predictive models of MU pool (a group of MUs that work together to coordinate the activation of a muscle) organization to analyze EMG generation [24]. MUs recruited earlier are in most cases smaller in size (i.e. the smaller neurons innervate fewer muscle fibers, yielding a smaller recorded action potential), commonly referred to as Henneman’s ‘size principle’ [25]. Additionally, in non-ballistic and sustained submaximal activations, higher-threshold MUs, which tend to be more fatigable than low-threshold MUs, usually have lower firing rates to reduce fatigue and support sustained motor output, defined as the ‘onion-skin’ [23, 26–28]. Thirdly, larger MUs are likely to have lower firing rates (‘rate-size association’) [29]. Notably though, these phenomena are gathered from data of intact muscle. Little investigation has examined relationships between MU features governing MU pool organization in residual muscle following amputation.

With lack of clarity on how amputation affects neuromuscular control of residual muscles at the MU-level, the specific goals of this study sought to test the validity of common assumptions in MU pool organization discussed above in residual muscle after amputation below the knee. We hypothesize that due to peripheral nerve and muscular damage in amputation, combined with disuse of residual muscles in passive prostheses, the size principle, onion-skin, and rate-size association are less organized in residual muscle (lower correlation) and compressed in modulation (flatter rates of change) compared to intact muscle. The results of this study may provide important knowledge that guides the future design and implementation of reliable model-based neural interfaces using EMG or MUs for robotic prosthetic limbs.

## 2. Methods

### 2.1. Subject recruitment

Six people with unilateral lower-limb amputation below the knee (BKA) (5 male, 1 female, 45.6 ± 16.7 years old) were recruited to participate in this study and provided written informed consent approved by the local institutional review board and in accordance with the Declaration of Helsinki. Table 1 displays more details on subject characteristics.

### 2.2. Experimental sessions

Subjects came in for two sessions up to a maximum of four hours separated by at least two days apart. The first session was dedicated to explaining the details of the study, acquiring informed consent, searching for appropriate locations for electrode placement on muscles of interest, and allowing subjects to practice controlling their muscle activations for both limbs using an interface providing visual feedback. All data used in analysis was collected in the second session.

### 2.3. Experimental setup

**Figure 1.**
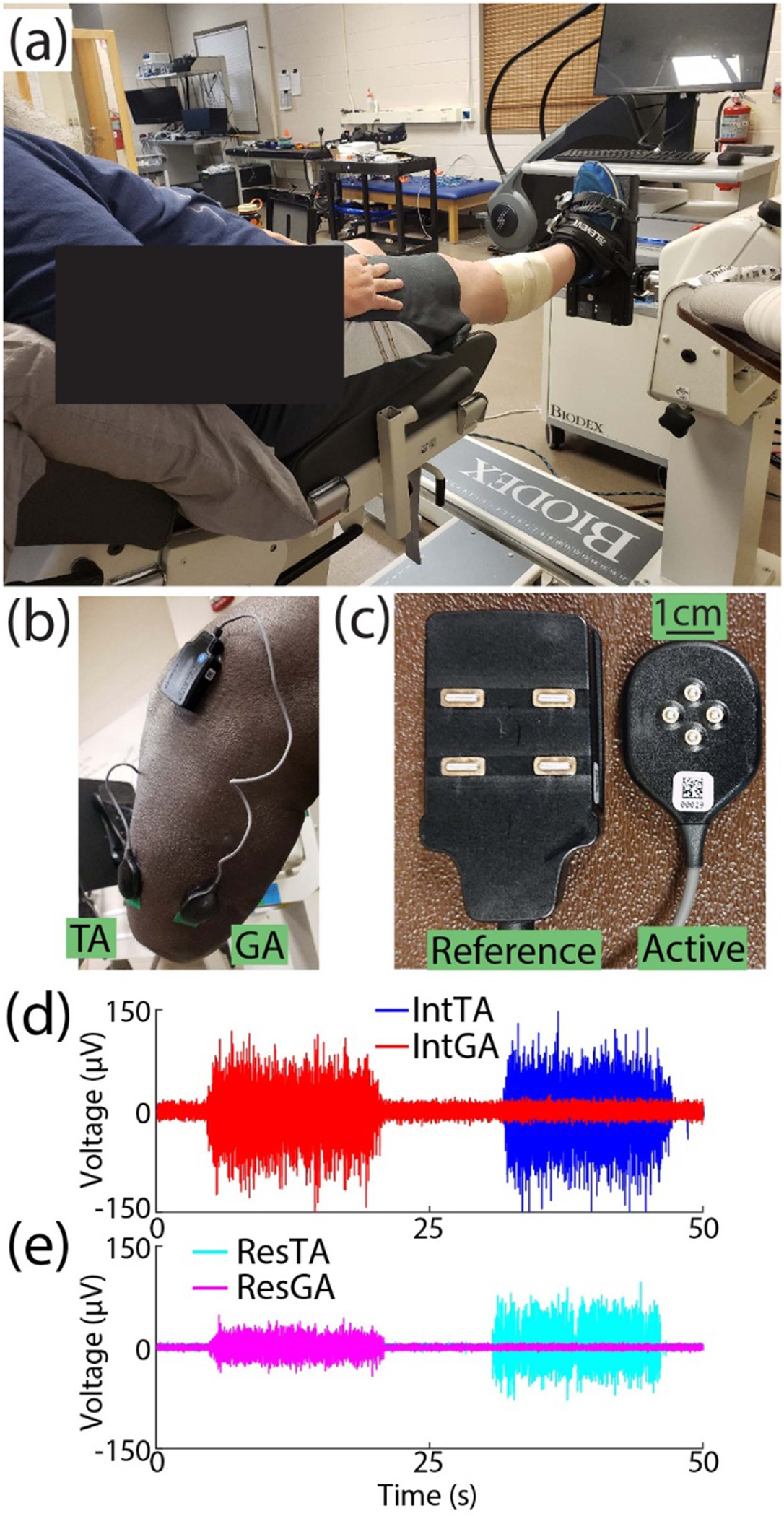
Experimental setup. (a) The ankle of intact limbs was fixed to a platform such that subjects conducted isometric muscle activation. (b) Example of electrode placement on residual limb TA and GA muscles. (c) Close-up of reference and active electrodes used for each muscle. Subjects alternated controlled activation of either their (d) intact TA (blue) and GA (red) muscles or (e) residual TA (cyan) and GA (magenta) muscles. Raw EMG from BKA3 is shown as an example.

EMG was recorded from two muscles of interest: the *tibialis anterior* (TA) and lateral *gastrocnemius* (GA) on both the residual (Res) and intact (Int) limbs. Prior to sensor placement, repeated palpation of each muscle was conducted to mark areas of interest. The area was cleaned with alcohol, 4-pin array electrodes (Galileo, Delsys, Inc., Natick, MA; figure 1(c)) were fixed to skin with a double-sided adhesive, and wrapped (MWrap, Mueller, Inc., Prairie Du Sac, WI) to provide additional pressure. Reference electrodes were placed on the lateral and medial tibial condyle nearby (figure 1(b)) [30]. Because all four channels for each are recorded as differential with a 5 mm inter-electrode distance, the small recording volume reduces crosstalk between muscles [31–33]. Still, to further mitigate this potential issue in the residual limb due to variation in muscle placement following surgery, following placement, EMG signals were inspected for crosstalk between the TA and GA while subjects alternated activation. We asked subjects to alternate activation of each muscle. If signals from both sensors visually appeared correlated while one muscle was targeted, the sensor for the non-targeted muscle was shifted up to approximately 1 cm to isolate activation of each muscle [34] and maintain signal-to-noise ratios above 2.5, which exceeded the device’s recommended minimum ratio by a factor of two to ensure high signal quality for decomposition.

Subjects sat in an experimental chair for all tasks. For the intact limb, the ankle was fixed to an attachment such that muscle activation was isometric (figure 1(a), System 4 Pro, Biodex, Shirley, NY), with the ankle at a neutral angle and the knee flexed at 5° to allow greater plantarflexion capacity [35] during experimental tasks (figure 1(d)). The residual limb rested off the chair to prevent extraneous muscle activity and minimize artifacts during residual limb tasks (figure 1(e)). The chest and thighs were strapped to further isolate task activity.

### 2.4. Experimental protocol

#### 2.4.1. Muscle activation calibration

After warming up with light muscle activity, subjects conducted two maximum voluntary contractions (MVCs) for each muscle (random order). Each MVC was 3 s with at least one minute of rest between MVCs. The MVC was then defined as the highest root-mean-square (RMS) in a 0.5 s window from a random channel. The higher of the two MVCs was retained; visual feedback of muscle activation using the same channel was then provided as a percentage of MVC by computing the RMS of the raw EMG in real-time (0.5 s window, full overlap) and dividing by the RMS computed during the MVC.

#### 2.4.2. Experimental tasks—profile tracking of muscle activation

Each limb was tested with the same experimental procedure (random order). For each limb, subjects conducted two trials targeting 20 and 35 %MVC (random order). These target levels were chosen after pilot testing targeting 10, 20, 30, and 40 %MVC at steady-state. 10 %MVC at steady state resulted in minimal activation, while 40%MVC was difficult for subjects to maintain and quickly led to fatigue. In the experimental session, subjects practiced the 20 %MVC level for each condition between two up to four times to re-familiarize with the interface and controlling targeted activation. Subjects did not practice the 35 %MVC target level in the experimental session to prevent fatigue.

In all trials, subjects were given simultaneous visual feedback of their TA and GA activation for the targeted limb. The target profile for each trial began with a 5 s quiescent period, followed by four repeated trapezoids, with a 10 s rest period between each trapezoid. Each trapezoid consisted of a 10 %MVC/sec ramp up to the targeted MVC level, which they held for a 12 s steady-state period, followed by a 10 %MVC/sec ramp down. For all trials, subjects sequentially alternated controlling activation of the TA and GA (first targeted muscle random), such that subjects controlled activation of each muscle two times in each trial. EMG data were sampled at 2,222 Hz (EMGWorks, version 4.7.9, Delsys, Inc., Natick, MA).

### 2.5. Data processing

#### 2.5.1. Decomposing and validating motor units

EMG data were filtered from 20 to 450 Hz [36] and decomposed via Neuromap Software (version 1.2.2, Delsys, Inc.), which uses a maximum a posteriori classifier that updates a library of MUAPs in order to minimize the difference between the measured EMG and a synthesized signal [22]. This technique has been independently validated with 2-source experimental testing [37] and rigorous analysis in simulation [38], and has been used in various applications [39–42]. For each MU, the decomposition outputs a MUAP recorded from each of the 4 channels, and a vector of time stamps when the MU fired. In addition to retaining MUs with accuracy greater than 90% accuracy by the algorithm output, as done in [39–42], valid MUs were retained via a 2-step spike-triggered averaging (STA) procedure described previously [29]. The first step averages raw EMG across all spikes in the trial in an 80 ms window centered at the spike time to isolate the MUAP in the EMG signal. This STA template and the decomposed MUAP template were normalized and cross-correlated for each channel using the MATLAB function *xcorr*, and the average cross-correlation across channels was computed. High cross-correlation values indicate the decomposed MUAP and spike trains were accurate. Additionally, assuming no electrode shift occurs, the recorded MUAP for a valid MU should be relatively stable over time. A moving window (8 s, 4 s step size) computed multiple STA templates in the trial. The coefficient of variation (CV) of the MUAP peak-to-peak amplitude (PPAmp) across all computed STA templates was calculated for each channel and averaged. MUs resulting in cross-correlation of MUAPs of at least 0.6 and segmented STA templates with CV of PPAmps less than or equal to 0.5 were retained for further analysis [29]. This validation procedure has been applied in multiple prior studies [43–45].

#### 2.5.2. Data analysis

Three MU property relations noted above (size principle, onion-skin, and rate-size association) were examined by analyzing three fundamental MU properties: recruitment threshold (RT), mean firing rate (MFR), and MU size (inferred via the decomposed PPAmp [29, 39, 46, 47]). The RT was defined as the average %MVC level in a 200 ms window of the EMG signal centered at the time a MU began firing at a rate of at least 5 pulses per second (pps). To compute the MFR, a 4 s window during each steady-state period of targeted muscle activation with the lowest CV of %MVC was found. As noted earlier, each trial contained two steady-state periods for each muscle. The MFR for MUs recruited in both steady-state periods were averaged between the two 4 s windows to associate a single MFR to a given MU.

The size principle, onion-skin, and rate-size association were examined by fitting models between RTs and PPAmps (linear), RTs and MFRs (linear), and PPAmps and MFRs (inverse power), respectively. Model coefficients of determination (*R*^2^) and coefficients of fitted slopes or decay exponents evaluated MU pool organization and modulation in each relationship, respectively. To further investigate potential relations among differences in MU recruitment and discharge patterns and visualize subject-specific differences, outcomes between the size principle and onion-skin were correlated. For each subject and muscle, the difference in *R*^2^ (Δ*R*^2^) from the intact limb to the residual limb was computed for each relation. The Δ*R*^2^ of the size principle was plotted against Δ*R*^2^ of the onion-skin. The same procedure was conducted for differences in fitted slopes (ΔSlope).

#### 2.5.3. Statistical analysis

TA and GA muscles were analyzed independently. To statistically compare MU organization and behavior for each of the three relationships, a mixed effect model was employed, with fixed effects of limb (intact, residual) and target %MVC (20, 35) and a random intercept (subject). Data were tested for normality with a Shapiro-Wilk test to determine use of a parametric versus nonparametric model [48]. For all statistical tests, *α* = 0.05.

## 3. Results

**Figure 2.**
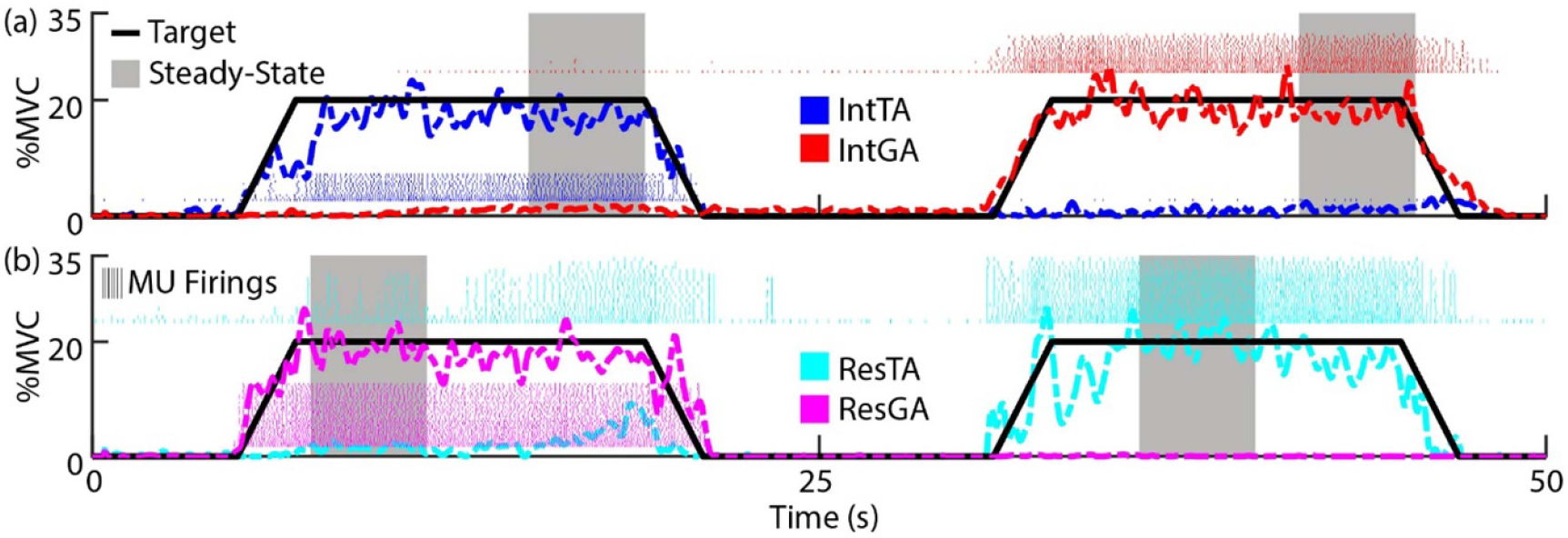
Representative tasks for each limb. Subjects were provided visual feedback of muscle activation of agonist/antagonist pairs from the same limb as a percentage of their respective MVCs. Subjects alternated controlling activation of each muscle by following a target profile (solid black line). BKA2 targeting 20 %MVC is displayed as an example. (a) Intact (Int) limb task, targeting the TA (blue) followed by the GA (red). (b) Residual (Res) limb task, targeting the GA (magenta) followed by the TA (cyan). In analysis, mean firing rates for each MU in a muscle were computed during steady-state regions (gray highlight). For visual brevity, the first half of the trial is displayed.

Figures 2(a) and (b) display the first half of representative tasks targeting 20 %MVC for the intact and residual limbs of BKA2, respectively. Subjects successfully modulated activation of each muscle in both intact and residual limbs following practice. Note that compared to similar studies using force tracking to control activation [23], there is greater variability due to use of EMG feedback in this study. During targeted activation of the ResGA (dashed magenta), non-targeted activation of the ResTA occurred during the second half of the steady-state activation period, which is expected, as coactivation in agonist-antagonist residual muscles has been examined in other studies [12]. However, the ResGA (in addition to IntTA and IntGA) were more isolated in activation. Overall, subjects were able to follow the target profile with less than 5%–7% root-mean-square error (see appendix) and maintain a steady-state level of targeted activation. Residual muscles tended to yield fewer MUs than intact muscles (see appendix), but all data for residuals of fitted property relationships were normally distributed permitting analysis of MU property relationships. The critical F-Statistic for all outcomes was 4.35.

### 3.1. Size principle

**Figure 3.**
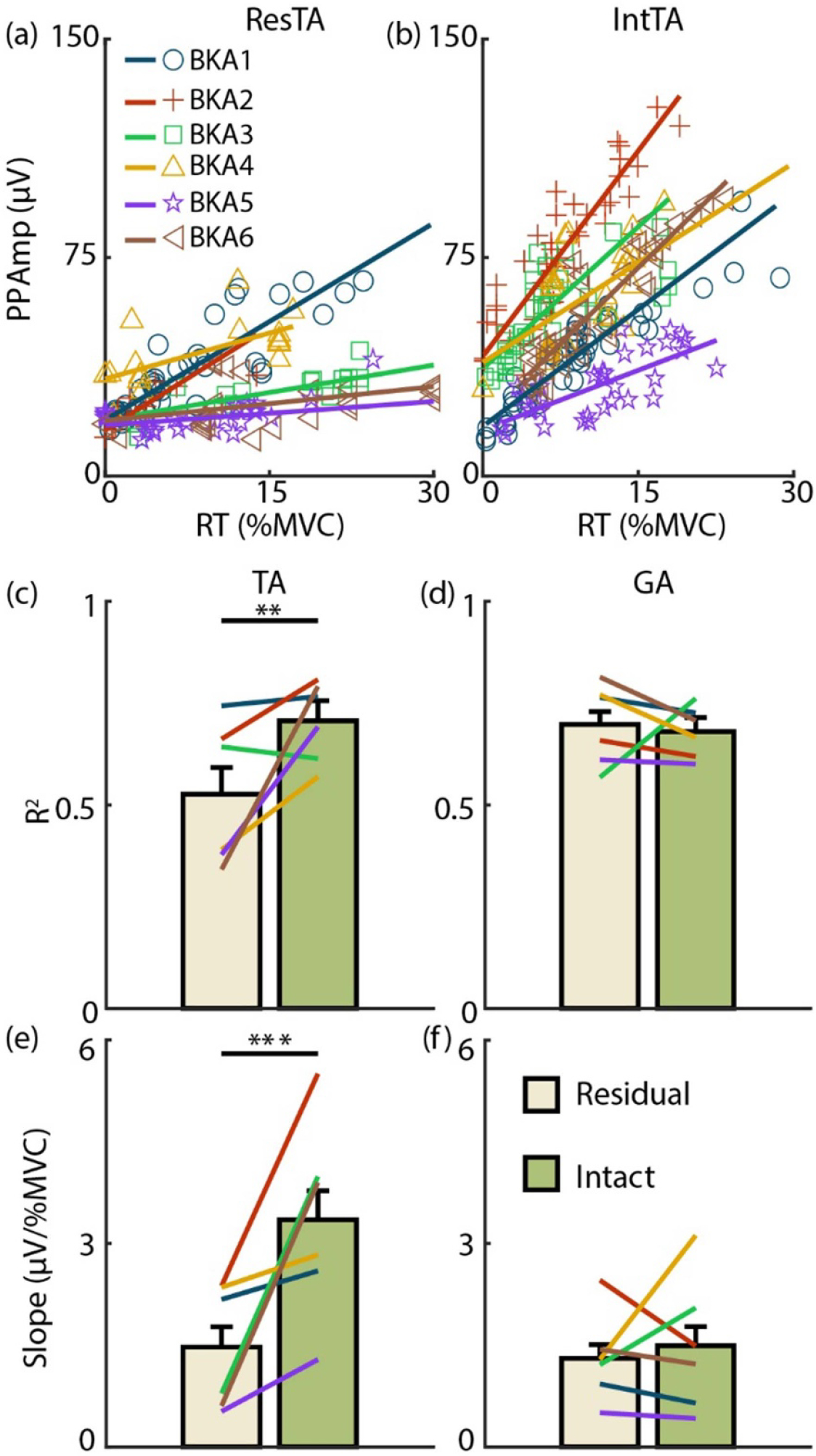
Size principle. Recruitment threshold (RT) is plotted against MUAP amplitudes (PPAmp) across subjects at 20 %MVC for the (a) Residual and (b) Intact TA as examples. Each marker is an individual MU. Solid lines indicate model fits for each subject. (c) and (d) Coefficients of determination (*R*^2^) for model fits for the TA and GA, respectively. Each bar shows the mean and standard error for the residual (light gold) and intact (green) limbs. Lines indicate subject averages across both MVC levels. (e) and (f) Slopes for each model fit, with the same structure as (c) and (d). ^*^p < 0.05, ^**^p < 0.01, ^***^p < 0.001 for all figures.

Figures 3(a) and (b) display size principle relationships across subjects targeting 20 %MVC for the ResTA and IntTA, respectively. In the IntTA, increasing RT consistently correlated with increases in PPAmp (i.e. MU size), and individual MUs fit tightly with the modeled linear regression. In the ResTA, PPAmp still increased with RT, but at a much slower rate. Figures 3(c) and (d) summarizes model fits in each limb across both %MVC levels for the TA and GA, respectively. The ResTA had significantly lower *R*^2^ (*F* = 9.28, *p* < 0.01) than the IntTA (mean ± standard errors (SEs) of 0.52 ± 0.07 and 0.71 ± 0.05 respectively), with all subjects exhibiting lower *R*^2^ in the ResTA, except BKA3, who exhibited slightly higher *R*^2^ in the ResTA. Alternatively, there was no significant difference in *R*^2^ for the GA (*F* = 0.16, *p* = 0.69, 0.70 ± 0.03 and 0.68 ± 0.04 for ResGA and IntGA, respectively), with only BKA3 resulting in a lower *R*^2^ for the residual limb. Figures 3(e) and (f) display fitted slopes for models in the TA and GA, respectively. The slope for the ResTA model was also significantly lower than the IntTA model slope (*F* = 26.0, *p* < 0.001, 1.5 ± 0.30 and 3.35 ± 0.43 *μ*V/%MVC, respectively), with all subjects exhibiting lower slopes in the residual limb. Again, no significant difference was observed in slopes between the ResGA and IntGA (*F* = 0.58, *p* = 0.46, 1.30 ± 0.21 and 1.50 ± 0.28 *μ*V/%MVC, respectively), with 4 of 6 subjects (all but BKA3 and BKA4) displaying similar or higher slopes in the residual limb.

### 3.2. Onion-skin

**Figure 4.**
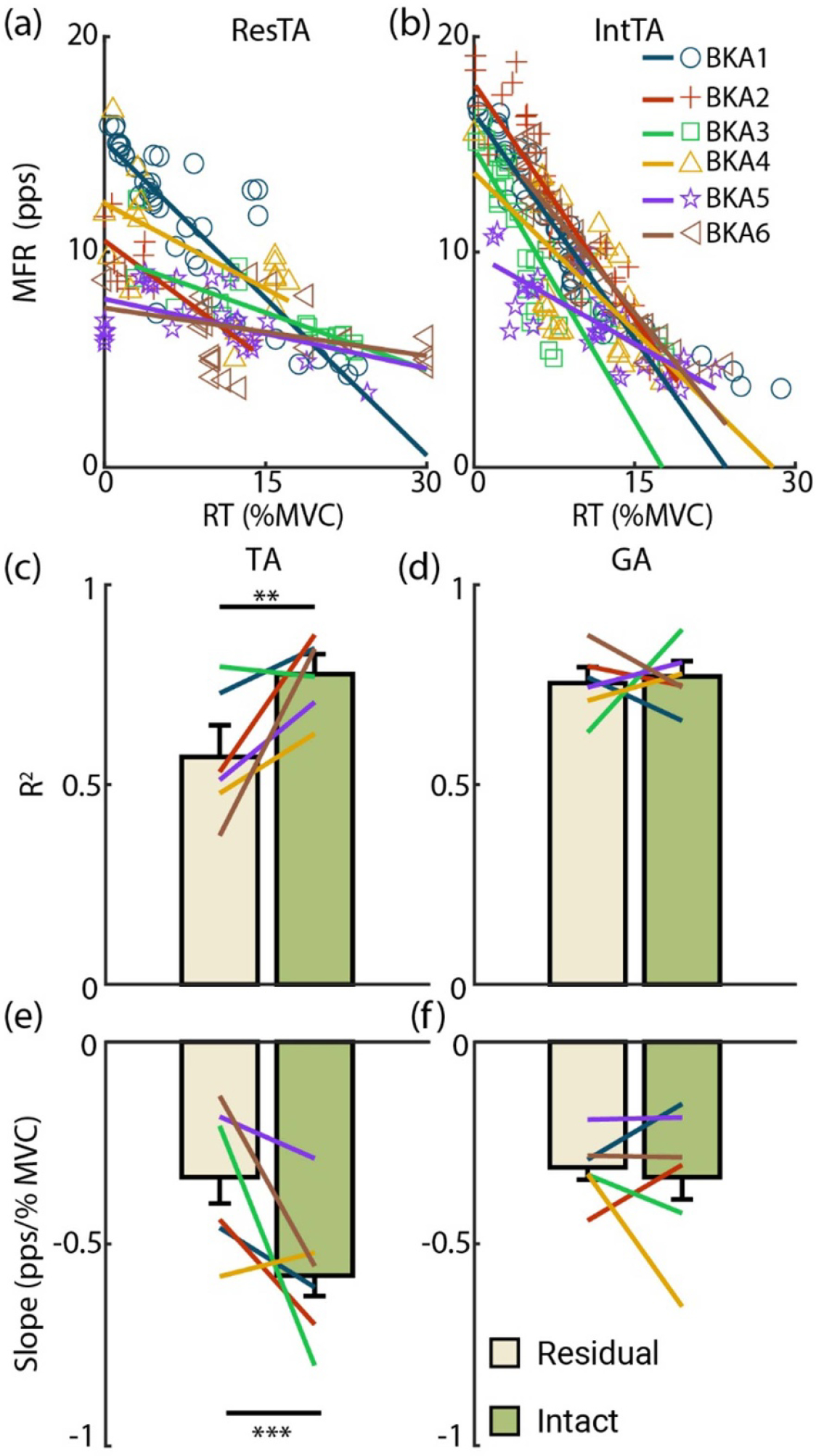
Onion-skin relationship. RT is plotted against mean firing rate (MFR) across subjects at 20 %MVC for the (a) residual and (b) intact TA as examples. (c)–(f) Have the same structure as figure 3.

A similar trend to the size principle was seen with the onion-skin for limb comparisons. In the examples of the ResTA (figure 4(a)) and IntTA (figure 4(b)) at 20 %MVC, MFR decreased with increasing RT in both limbs. In the IntTA, intersubject variability was low, with similar slopes and little scatter of MUs relative to model fits. Greater intersubject variability in fitted slopes and scatter of MUs was observed in the ResTA, but intrasubject comparisons were consistent for the majority of subjects. Across all subjects except BKA4, MFR tended to decrease at a slower rate compared to the IntTA. Indeed, the ResTA had significantly lower *R*^2^ (*F* = 10.03, *p* < 0.01, 0.57 ± 0.08 and 0.78 ± 0.05), with only BKA3 exhibiting similar *R*^2^ values between limbs (figure 4(c)). Subjects had significantly higher (flatter) fitted slopes for the ResTA than the IntTA (*F* = 14.83, *p* < 0.001, −0.34 ± 0.07 and −0.58 ± 0.05 Hz/%MVC), with only BKA4 exhibiting lower slopes in the ResTA (figure 4(e)). In the GA, only BKA3 had a large drop in *R*^2^ in the residual limb (figure 4(d)), and both BKA3 and BKA4 had flattened slopes (figure 4(f)). Across subjects, no significant differences were observed between the ResGA and IntGA for either *R*^2^ (*F* = 0.58, *p* = 0.46, 0.75 ± 0.04 and 0.77 ± 0.04) or fitted slopes (*F* = 0.26, *p* = 0.61, −0.31 ± 0.03 and −0.34 ± 0.06 Hz/%MVC).

### 3.3. Rate-size association

**Figure 5.**
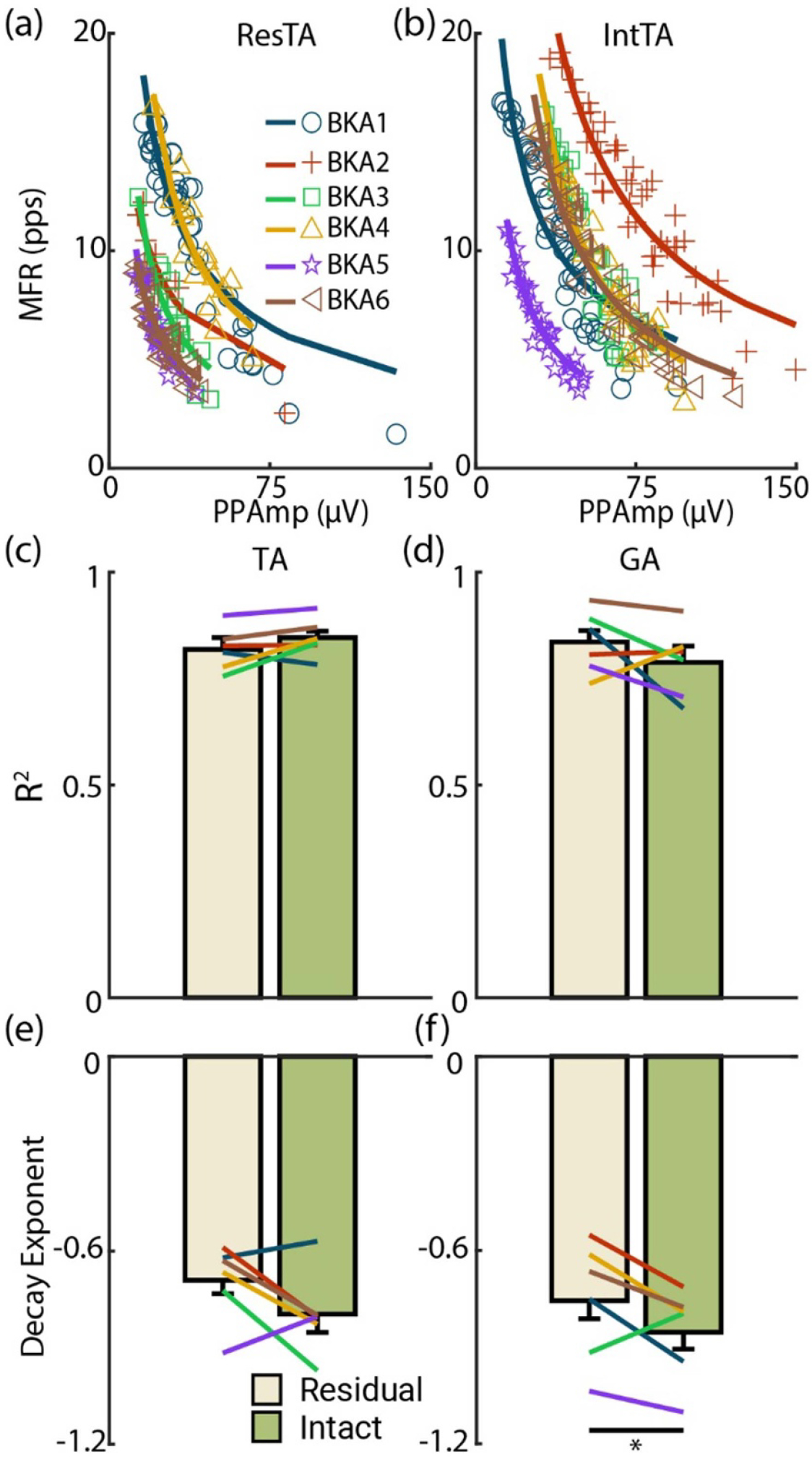
Rate-size association. PPAmp is plotted against MFR across subjects at 20 %MVC for the (a) residual and (b) intact TA as examples. (c)–(f) have the same structure as figure 3

Markedly, unlike the size principle and onion-skin, rate-size relationships were strongly correlated in both limbs and muscles (figure 5). In the ResTA (figure 5(a)) and IntTA (figure 5(b)) at 20 %MVC, there was intersubject variability in MU properties, but within each subject, the fitted model tightly followed the scatter of respective MUs. In the TA, no significant difference in *R*^2^ was observed between residual (0.81 ± 0.028) and intact (0.84 ± 0.016) limbs (*F* = 1.18, *p* = 0.29, figure 5(c)). In the GA, the average *R*^2^ for the residual limb (0.84 ± 0.027) was slightly higher than the intact limb (0.79 ± 0.039), but no significant difference was observed (*F* = 1.38, *p* = 0.25, figure 5(d)), with only BKA4 displaying a slight drop (0.82 intact to 0.74 residual). For the decay exponent, a higher (lower magnitude) value indicates increases in PPAmp yielded comparable MFR. The ResTA had a higher exponent (−0.69 ± 0.042) than that of the IntTA (−0.80 ± 0.057, *F* = 3.77, *p* = 0.067, figure 5(e)), with all but BKA1 and BKA5 displaying higher decay rates in the residual limb. The GA results were similar (figure 5(f)), with decay in ResGA (−0.76 ± 0.057) significantly higher than that in IntGA (−0.86 ± 0.053, *F* = 4.70, *p* = 0.042), and all but BKA3 having higher decay rates in the residual limb.

### 3.4. Interaction and %MVC level effects

No significant interactions between limbs and target %MVC levels were observed, except for *R*^2^ of PPAmp predicting MFR in the TA (*F* = 7.27, *p* < 0.05). In this condition, no significant main effects were observed (figure 5(c)). Based on these results, data shown (figures 3–5) combined model fit results across targeted %MVC levels to show limb effects as the primary research question.

### 3.5. Size principle and onion-skin correlations

**Figure 6.**
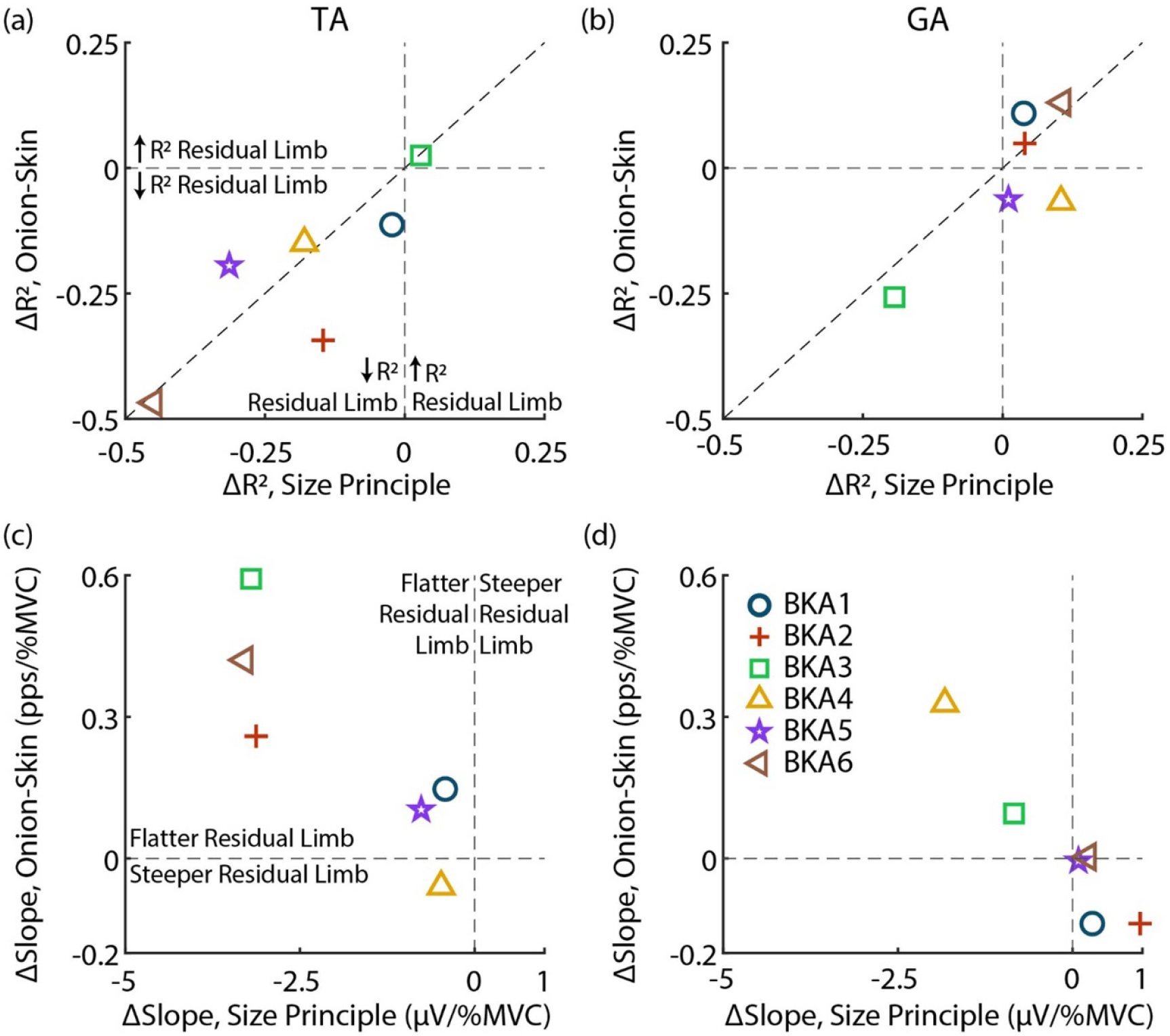
Relative change between size principle and onion-skin for each subject. (a) and (b) Change in *R*^2^ (Δ*R*^2^) for the TA and GA, respectively. Vertical and horizontal lines indicate no change in the size principle and onion-skin, respectively. The diagonal displays an identical change in both relations. (c) and (d) ΔSlopes for the TA and GA. In all cases the intact limb is the reference (residual minus intact).

Figure 6 displays relative changes in the size principle and onion-skin for each subject. As observed in figures 3 and 4, MU organization (*R*^2^) is disrupted in the TA for both relations (figure 6(a)). All subjects, except for BKA3 (0.029 Δ*R*^2^ size principle, 0.025 Δ*R*^2^ onion-skin), are in the lower-left quadrant, indicating lower *R*^2^ in the residual limb for both the size principle and onion-skin. Besides BKA1 (−0.023 Δ*R*^2^ size principle, −0.11 Δ*R*^2^ onion-skin), who was least impacted aside from BKA3, subjects who had a larger drop in *R*^2^ for the size principle also tended to have a greater reduction in *R*^2^ for the onion-skin (plotted near the diagonal). Organization of the GA (figure 6(b)) was less impacted, with most subjects clustered near 0 Δ*R*^2^ for both relations except for BKA3, who had a similar drop in *R*^2^ for both relations (−0.19 Δ*R*^2^ size principle, −0.26 Δ*R*^2^ onion-skin).

Comparing relative changes in modulation (fitted slopes), similar to Δ*R*^2^ observed, subjects who had flatter slopes in the residual limb for the size principle also had flatter slopes for the onion-skin in the TA (figure 6(c)). BKA4 (−0.48 *μ*V/%MVC, −0.059 pps/%MVC) was the only subject without flatter slopes in the onion-skin for the ResTA, with little ΔSlope for the size principle. BKA1 (−0.42 *μ*V/%MVC, 0.15 pps/%MVC) and BKA5 (−0.77 *μ*V/%MVC, 0.10 pps/%MVC) were also clustered closer to 0 ΔSlope for both relations. BKA2 (−3.13 *μ*V/%MVC, 0.26 pps/%MVC), BKA3 (−3.20 *μ*V/%MVC, 0.59 pps/%MVC), and BKA6 (−3.3 *μ*V/%MVC, 0.42 pps/%MVC) had similar ΔSlope for the size principle but different ΔSlope for onion-skin. In the GA (figure 6(d)), 4 of 6 subjects had no flattening of slopes in residual limb. BKA3 (−0.84 *μ*V/%MVC, 0.10 pps/%MVC) had a slightly lower slope in the residual limb in both relations, and BKA4 (−1.8 *μ*V/%MVC, 0.33 pps/%MVC) had the largest flattening of slopes for both relations across subjects.

## 4. Discussion

This study characterized fundamental relations in MU pool organization between intact and residual muscles after amputation below the knee. Many studies have investigated how disruptions to the central nervous system (as seen in survivors of stroke [49], individuals with incomplete spinal cord injury [50], and other diseases [51, 52]) can disrupt the peripheral neuromuscular system and EMG signals. Alternatively, our experiment is one of the first to show disruption of the peripheral neuromuscular system (through limb amputation) leads to changes in central neuromuscular control. We used a validated technique decomposing MUs from EMG to evaluate changes in MU pool organization within a muscle. Specifically, we observed the size principle and onion-skin were significantly disrupted in organization and had compressed modulation in the ResTA, but these relationships were not significantly impacted in the ResGA. The rate-size association had compressed modulation in both muscles for most subjects, but organization of this relationship was preserved. We showed not only does limb amputation affect MU properties at the periphery (such as MU size), but peripheral damage also causes spinal-level functional reorganizations. Specifically, our overall findings suggest systematic muscle-specific interference occurs to recruitment and discharge patterns of the MU pool following transtibial amputation.

### 4.1 . Size principle

In intact muscle, earlier recruited MUs tend to be smaller in size [25]. We observed this relationship is significantly less correlated and flattened in the residual TA after amputation. This disorganization could be caused by multiple factors. First, residual muscle exhibits significant loss of cross sectional area (atrophy) following amputation [53]. It is possible atrophy occurred from either muscle fiber size reduction or loss in amputation surgery. Indeed, the ResTA (figure 3(a)) exhibited a distinct reduction in the distribution of PPAmp (*y*-axis) across subjects compared to the IntTA (figure 3(b)), suggesting atrophy or muscle fiber loss has compressed the size principle. In parallel, if input resistance at the dendrites and soma of corresponding motor neurons was maintained, the RT would be unaffected [54]. If a nonuniform effect across the MU population occurred, this would lead to disorganization in the size principle, and would also explain the flattened slopes we observed. BKA6 had the largest reduction in *R*^2^ and slope for the size principle and was the youngest among those tested at the time of their amputation (11 years-old). In addition to atrophy and fiber loss, amputation may have stunted development of muscle fibers, further amplifying differences observed from the intact muscle. Besides these factors affecting muscle fibers, it is possible reinnervation impacted our findings. Reinnervation of smaller MUs can be more effective than that of larger MUs [55], which after amputation could lead to recruitment reversals, where lower-threshold MUs increased in size relative to larger MUs, further contributing to lower correlations and flattened slopes observed.

Alternative to relative changes in MU size disrupting the size principle, it is possible relative changes in RT between MUs occurred. Although Ia afferents are widely distributed in intact muscle [56, 57], regeneration following nerve injury [58] due to amputation may change their distribution and activity, which could disrupt RTs [59]. Direct studies on Ia afferent activity in residual muscle are needed determine potential effects on RTs. Interestingly, multiple subjects noted ‘lack of pressure’ made it difficult for them to control their muscle activation to the same degree as their intact muscle, which was provided passive cutaneous feedback from the footplate during isometric activation. Cutaneous stimulation can result in recruitment reversals between high- and low-threshold MUs [60]. Hence, disruption to cutaneous afferents could instead contribute to recruitment reversals, bringing disorder to the size principle. Loss of cutaneous afferent input can also result in deficits of muscle activation [61]. If EMG amplitude was reduced with loss of afferent input, observed MU sizes would also lower, resulting in flattened slopes observed. Future studies analyzing the effect on MU properties when providing artificial cutaneous feedback to the residual limb [62], or preserving feedback of Ia afferents [63], may shed light on these hypotheses.

### 4.2. Onion-skin

In intact muscle, MU MFR usually reduces with increasing RT [26]. For the TA, MUs in residual muscle were less organized compared to MUs of intact muscle. RT reversals of MUs discussed above could have affected not only the size principle but also the onion-skin. If excitatory drive and firing rates for MUs were not impacted by amputation, and RTs of MUs reversed, correlations between the RT and MFR would reduce. Additionally, recruitment reversals alone could explain compression in rate modulation that we observed. For the onion-skin of intact muscle, higher-threshold MUs have lower ranges of excitation (after recruitment) driving increases in firing rates compared to lower-threshold units [26]. If higher-threshold MUs are recruited earlier in activation, they would produce higher firing rates, flattening the relationship. Disorder observed directly indicates spinal-level functional reorganization in residual muscle. BKA2 and BKA6 had the largest reduction in *R*^2^, and both were adolescents at the time of amputation (11 and 15 years old, respectively), suggesting greater reorganization may have occurred after surgery in these subjects. Additionally, reduction in neural drive, potentially from prolonged disuse of the muscle [64], would compress the range of excitation to the MU pool and range of MFRs. Future comparisons of the magnitude of common synaptic inputs to the MU pool of each muscle may shed light on the extent to which neural drive [65] and afferents in spinal circuits [66] may differ between intact and residual muscle.

### 4.3. Rate-size association

Our findings suggest the relationship between MU size and firing rates of the MU pool is preserved. Distinct to the size principle and onion-skin, no significant difference was observed in organization (correlation) of the rate-size relationship in any muscle, and high *R*^2^ values were maintained (⩾0.79). The experimental task goal was to control muscle activation with the target line, including a steady-state period of increased activation. Increases in MU sizes recruited, causing the EMG signal to increase, may have been balanced with MFR decreases to maintain stability of sustained muscle activation, as subjects were able to reasonably follow the experimental task (figure 2). Still, for 4 of 6 subjects the decay exponent was higher in the residual limb for both muscles, suggesting smaller changes in MFR occurred in residual muscle with increasing MU size. Considering the exceptions, BKA1 was an outlier in this comparison, with a much flatter decay (−0.57) in the IntTA compared to the average (−0.80 ± 0.057). BKA5 had distinctly compressed ranges of MFR in their IntTA compared to other subjects (figure 5(b)), which would flatten decay, potentially due to aging [67], as they were the only older-adult recruited (67 years-old). Because the residual muscle was amputated nine years prior, aging effects flattening decay may not have occurred in residual muscle no longer used, leading to sharper decay in the residual limb. Flattened decay in residual limbs exhibited by the majority of subjects can arise from mechanisms mentioned above. Potential muscle fiber loss, reducing size of the MU pool, would have compressed both the size principle and the rate-size association. Additionally, just like the onion-skin, earlier excitation of high-threshold MUs in recruitment reversal, or reduction of overall neural drive, could compress the observed range of MFRs and flatten decay. The primary distinction between the rate-size association and the two former relationships discussed is it does not involve the RT. With the rate-size organization preserved, and similar effects on the residual limb observed between the size principle and onion-skin (figure 6), it is possible disorder in RT may be most severely impacting the size principle and onion-skin relative to disordered changes in MFR and MU size. Future coherence analyses to infer common synaptic inputs to MU pool discharges in residual muscles are necessary to shed light on this hypothesis.

### 4.4. Muscle-specific effects

Notably, among relationships analyzed, the TA was consistently impacted in both organization and modulation. However, except for significant flattened decay rates for the rate-size association, the GA was not as impacted. Thus, it is important to consider muscle-specific outcomes observed. A strong possibility is the TA may be more sensitive to changes in neural inputs. First, reflex amplitudes in reciprocal inhibition are significantly higher in the TA relative to the *soleus* and medial *GA*—other plantarflexors within the triceps surae like the lateral *GA* studied here—suggesting afferent inputs into this muscle are larger in magnitude [68]. Because efferent-afferent neural pathways are highly integrated [69], any disruption to afferents due to amputation may have had a larger effect in the TA for efferent neurons we have studied. Additionally, persistent inward currents (PICs), correlated to monoaminergic inputs that regulate intrinsic neuromodulation, are significantly larger in magnitude in the TA compared to the *soleus* [70]. Explicit studies are necessary to determine if PICs differ in residual muscle. However, since cortical reorganization is often observed following amputation [71] and linked to long-term changes in GABA function (a common monoamine inhibitor) [72], it is possible that deviations in neuromodulation following amputation produce an asymmetric down-stream effect on the MU pool. Furthermore, coherence of muscle activity in the beta band, commonly associated with the magnitude of cortical neural inputs [73], is significant in the TA but not in the GA during both isometric and isotonic activity [74], suggesting any cortical reorganization would maintain a larger impact on the TA. Although causal effects cannot be concluded from our observations, we hypothesize direct changes to neural behavior centrally and peripherally following amputation play a large role in our findings.

Beyond changes in neural dynamics, structural factors should also be considered. First, the *GA* is a biarticular muscle that crosses both the ankle and knee, while the *TA* only crosses the ankle, which could explain why a smaller bilateral difference was observed in the GA compared to the TA. Considering surgical factors, conventional lower-limb amputation surgeries have not significantly changed in decades. Among techniques, the long posterior flap [75] is the most commonly employed [76]. In this procedure, the incision disrupts the anterior portion of the limb more proximally than the posterior side. The anterior muscle compartment is entirely or partially (the case for subjects in this study who may have had this procedure) excised, while the GA is thinned only as necessary to cover the tibial bone [76]. Additionally, humans consistently have a proximal (10.5 ± 1.6 cm distal to the apex of the fibular head) and distal (16.5 ± 1.9 cm distal to the apex) motor point in the TA, with only one motor point in the GA (9.8 ± 2.3 cm distal to the apex) [77], suggesting there may be bimodal distribution in the density of motor end plates in the proximal-distal direction for the TA. While speculatory due to lack of data on surgical details and neural or muscle architecture for subjects in this study, it is possible some subjects’ surgery led to a larger structural disruption on the TA compared to the GA, or there was an asymmetric disturbance to neuromuscular junctions of the MU pool in the TA. If the innervation zone for a MU is lost, MU sizes would reduce (lowering MUAP amplitudes recorded), while also disturbing organization of the motoneuron pool. As efforts redesigning amputation surgery continue to grow [78], comparing techniques directly with musculoskeletal architecture and MU properties together may improve patient outcomes, especially for those who desire myoelectric prostheses.

### 4.5. Implications for EMG modeling

The primary motivation for our study was to test the common assumption that properties of EMG generation in residual muscle are similar to intact muscle. Based on our results, this assumption does not always hold. In simulations of EMG, predictive computational models of MU pool organization are often used to represent neural commands. In a commonly employed model of EMG and force generation [24], MU firing rate linearly increases with excitation above the RT. To account for disorder in the relationship of the onion-skin we observed, either an element of noise should be added into this function, or a nonlinear model between recruitment and firing rates may be more accurate. Additionally, peak firing rates, modeled inversely proportional to the RT, are based on the difference in peak firing rate from the first to last MU recruited. If the onion-skin is compressed, the difference in peak firing rates across the MU pool should be lowered. The twitch force for the MU is also modeled by the RT in accordance with the size principle. Based on disorder we observed, the peak twitch force for a given MU should have added noise, and the range of peak forces could be reduced to account for compression in the size principle.

While generalizable models of MU relations are ideal, BKA make up a heterogeneous population exhibiting high variation in muscle activation patterns [79]. In our study, of the 12 comparisons conducted between intact and residual limbs (two outcomes, three MU relations, two muscles), all had at least 4 of 6 subjects follow the same trend, but only two comparisons (size principle slope for TA, figure 3(e), and rate-size *R*^2^ for TA, figure 5(c)) elicited the same result for all six subjects. Across subjects, BKA3 (green square) particularly stood out, opposing the observed trend in 7 of 12 comparisons, spanning outcomes in all three MU relations and both the TA and GA (figures 5(f), 6(a), (b) and (d)). Compared to other subjects, BKA3 anecdotally was by far the most active subject (hikes 3–5 km on unpaved trails almost daily). Knowing MU properties can be plastic, as peak firing rates can reduce after immobilization [80] or increase with targeted training in older adults [81], and MU size can increase after strength training [39], this activity level may have supported higher organization (*R*^2^) and modulation (slope) of motor control on their intact limb, leading to larger differences observed in the residual limb, given these muscles are not used in passive devices. For example, they had the second-highest slopes on their IntTA and IntGA for the size principle (figures 3(e) and (f)), the highest onion-skin *R*^2^ in the IntGA (figure 4(d)), and the lowest and second-lowest slopes in the onion-skin for the IntTA and IntGA, respectively (figures 4(e) and (f)), giving a much larger range for potential disorganization and flattened slopes with their residual limb. Moving forward, caution must be taken when using default parameterization of MU organization to simulate residual muscle EMG, particularly because both muscle- and subject-specific disruptions to MU pools can occur.

### 4.6. Implications for continuous myoelectric control

Our results also relate to outcomes observed in EMG-driven musculoskeletal models intended for myoelectric prosthesis control. In a prior experiment, subjects balanced a virtual inverted pendulum with applied torque in each direction proportional to TA and GA muscle activation while sitting with a passive prosthesis donned [34]. Subjects had much higher rates of dropping the pendulum in the direction of TA activation, with significantly higher torque applied for the same change in angle, giving less resolution of pendulum control. However, ResGA control was similar to GA control by people without amputation. In that study, deficits in ResTA control were associated with qualitative visual observations of greater skin deformation during ResGA muscle activation compared to deformation during ResTA muscle activation. With this, subjects may have been provided more cutaneous feedback within the prosthesis socket due to higher pressure against the socket lining. It is true the popliteal depression region, which neighbors the GA, exhibits greater pressure compared to the lateral tibial area, closer to the TA, during daily activities [82]. Additionally, the popliteal depression exhibits much higher pressure sensitivity to increases in tissue stiffness that would occur in muscle activation [83]. Cutaneous feedback from the socket indeed may have supported better control of the GA. However, in our study, no cutaneous feedback was provided to residual muscle, and we still observed more maladaptive effects in the TA compared to the GA. Based on our results, it is possible disorder and compression of the size principle and onion-skin may contribute to worse outcomes in direct EMG control. The EMG envelope is generated from the number and size of active MUs in combination with their respective firing rates. If the size principle and onion-skin are compressed, resolution of changes in EMG amplitude would lower, leading to the larger normalized applied torque that was observed in the ResTA. Additionally, disorder in the relationship would lead to greater variation in the envelope and more difficult continuous control.

Considering this hypothesis, a year prior to the current study, two subjects also participated in a training protocol with a powered prosthetic ankle using direct EMG control of pneumatic muscles to improve postural stability [6]. BKA4 used a strategy invoking primarily ankle excursion to maintain balance, while BKA1 employed a mixed strategy invoking hip, knee, and ankle excursions to maintain balance, suggesting BKA4 may have had greater resolution of direct EMG ankle control. Interestingly, in our study, BKA4 was the only subject with similar slopes of the onion-skin between the ResTA and IntTA, and BKA1 was also clustered near zero change in *R*^2^ and slopes (figure 6(c)). However, BKA4 had flatter size principle and onion-skin slopes for the GA compared to other subjects (figure 6(d)), suggesting compensatory behaviors may also occur to maintain direct EMG control of the ankle. It is possible BKA1 and BKA4 had surgical procedures that preserved the size principle and onion-skin of their ResTA muscles. Alternatively, as mentioned above, training the often unused residual muscle may have increased peak firing rates [80, 81] or MU size [39] in residual muscles. With direct EMG control, a case study by our group has indeed shown common drive between the ResTA and IntTA for interlimb coordination increases after training [84]. Rather than preservation in surgery, recent practice controlling residual muscles may have *restored* MU relations for these individuals. It would be highly interesting to characterize MU property relationships during training with a continuous myoelectric controller in either a virtual or real-world environment in the future. If performance correlates with restoration of the size-principle or onion-skin, MU pool adaptations could be used as physiologic-based biomarkers to inform and potentially accelerate rehabilitation. For example, if the size principle and onion-skin were impacted by surgery, extra training in a virtual environment practicing neuromuscular control may be needed to improve functional potential. If these relationships were not disrupted, as seen with the TA for BKA4, they may be ready to train with a prosthesis earlier. As dynamic MU decomposition [85] and built-in electrodes inside prostheses [86] advance, characterizing MU behavior in functional tasks may clarify explanations for myoelectric controllers’ often worse performance in people with amputation relative to people without amputation [87]. Overall, with the promise that internal models of motor control are adaptable through training [88], rehabilitative protocols targeting potential deficits in neuromuscular control could be implemented to improve functional performance with myoelectric prostheses.

### 4.7. Limitations

There were limitations to this study that must be noted. First, steady-state activation levels up to 35 %MVC may have biased sampling to lower-threshold MUs. Given that MUs are often recruited in an exponential pattern with excitation [24], we expect MUs analyzed are representative of a large portion of the MU pool. Still, comparisons at higher activations are necessary to determine if phenomenon such as reverse-onion skin profiles can also occur in residual muscle [89]. Additionally, this work does not provide exact recommendations for changes in parameterization to EMG models. Instead, we tested current assumptions of MU property relationships, and found these assumptions are not always valid. More testing is needed to provide generalizable, robust recommendations of changes to models of MU pool organization for residual muscle, and potential interactions with other demographic factors such as age and time since amputation. Technical limitations must also be considered. Extraneous adipose tissue in residual muscle could have filtered EMG and reduced MU sizes recorded. There can be a higher percentage of adipose tissue infiltrating residual muscle compared to intact muscle, but large intersubject variability exists [90]. Studies measuring tissue composition with MU behavior are needed to determine if this effect is significant compared to other factors discussed.

## 5. Conclusion

To the authors’ knowledge, this study is one of the first to show that peripheral damage to the neuromuscular system disrupts fundamental relationships of MU recruitment and discharge patterns. Our outcomes suggest current models for MU pool organization may lack validity in residual muscle after amputation. Further study is needed to realize mechanisms generating MU pool reorganization and update parameterization to improve accuracy of predictive MU pool models generating EMG. In the long-term, refining understanding of MU pool organization after amputation could inform design of musculoskeletal model-based myoelectric controllers better representing residual limb physiology and support effective training for myoelectric prostheses.

## Figures and Tables

**Table 1.** Subject characteristics.

Subject ID	Sex	Age (Years)	Height (cm)	Mass (kg)	Time since amputation (Years)	Cause of amputation

BKA1	M	53	174	103	6	Trauma
BKA2	M	19	180	83.3	4	Trauma
BKA3	M	52	196	119	3	Intractable Pain
BKA4	M	42	183	99.3	19	Trauma
BKA5	F	67	165	74.4	9	^a^PAD
BKA6	M	30	175	122	19	Trauma

Mean (SD)	—	43.8 (17.3)	179 (10.4)	100 (18.9)	10 (7.27)	—

a Peripheral arterial disease.

**Figure 7.**
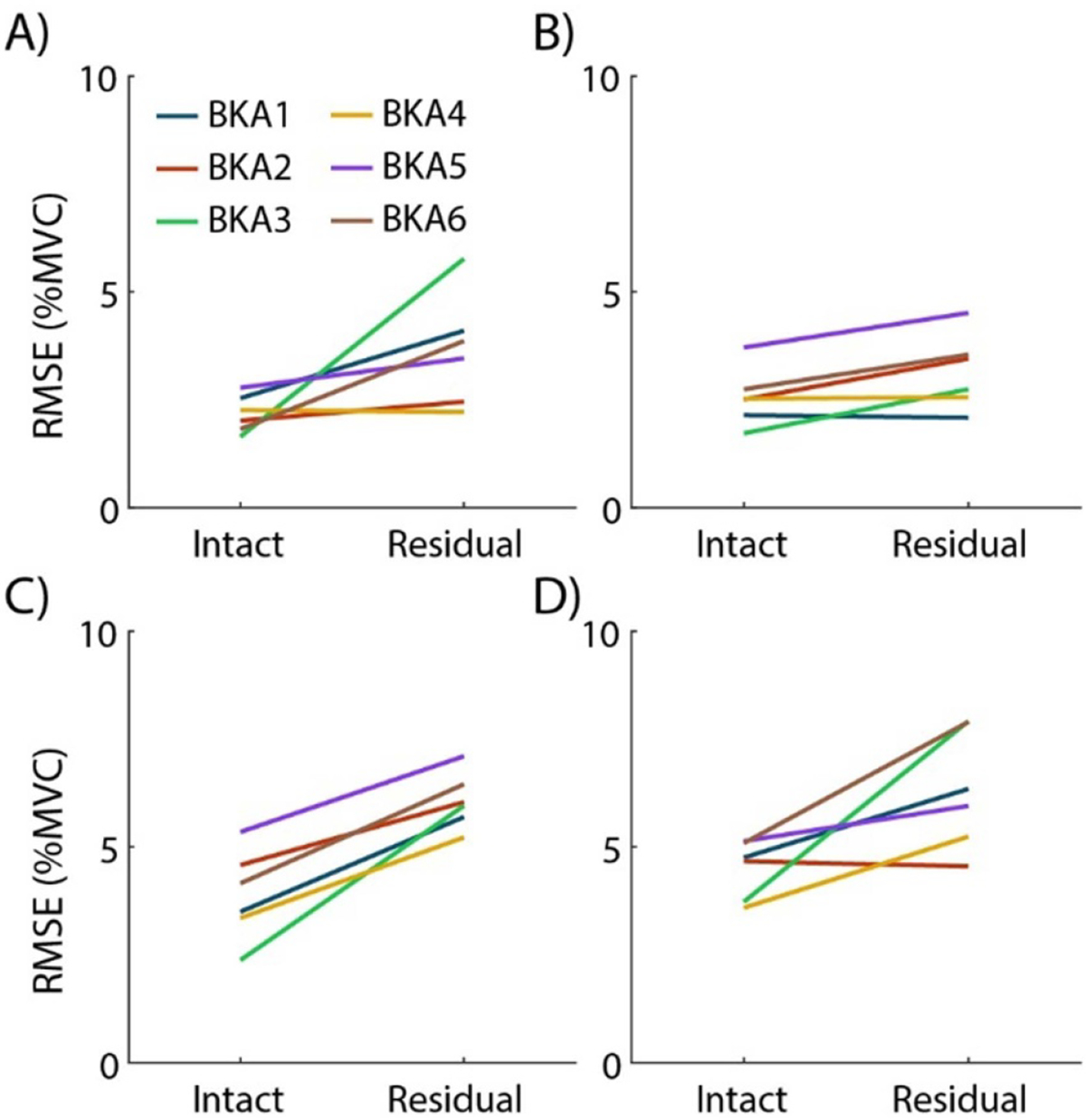
Root-mean-square error (RMSE) between actual activation and the target profile traced. Average error for each subject is shown for the (a) TA, 20 %MVC, (b) GA, 20 %MVC, (c) TA, 35%MVC, and (d) GA, 35 %MVC conditions.

## Data Availability

The data that support the findings of this study are available upon reasonable request from the authors.

## Appendix

### Profile tracking performance

To quantify subjects’ ability to follow the target profile, the average RMSE to the target is displayed in figure 7. For all four conditions, subject errors ranged from 2 to 7%MVC error. Residual limbs tended to have slightly higher user error, which is expected due to disuse of these muscles in passive devices prior to the study. A justifiable threshold for matched muscle activation between limbs to our knowledge has not been conducted in prior studies, potentially due to an upper limit of minimizing user error. We believe these errors are reasonable within the scope of our experimental paradigm.

### MU yield and individual MU properties

Table A1 shows averages and standard errors for MU yield and individual MU properties used to study the relationships in the MU pool. Residual muscles tended to yield fewer MUs, but residuals of fitted models were normally distributed, allowing comparison of models fitted to intact versus residual muscles. Differences in RTs exhibited high variability across subjects. Peak-to-peak amplitudes of MUAPs were consistently lower in the ResTA compared to the IntTA, which aligns with flattening of slopes in the size principle (figure 3(e)). Mean firing rates tended to lower in the ResTA compared to the IntTA, but interpretation of this difference in terms of differences in excitability must be taken with caution. Absolute muscle activation cannot be perfectly matched between muscles due to user error, as seen in figure 7. Because of this, rather than comparing direct MU properties, the main paper above studied well-established relationships between recruitment and discharge patterns across the MU pool to elucidate changes in mechanisms of neural activation in residual muscle.

**Table A1.** Summary of MU characteristics.

Property	Subj	ResTA	IntTA	ResGA	IntGA

	BKA1	23.8 ± 1.9	32.5 ± 4.8	21.3 ± 4.9	31.5 ± 4.4
	BKA2	15.8 ± 5.4	25.0 ± 2.0	17.8 ± 2.2	15.8 ± 1.3
# MUs	BKA3	8.8 ± 1.3	20.3 ± 2.8	14.5 ± 2.8	18.5 ± 3.8
	BKA4	11.3 ± 5.1	18.3 ± 2.9	25.8 ± 1.1	21.8 ± 2.5
	BKA5	11.5 ± 1.8	19.0 ± 3.0	10.5 ± 2.4	12.3 ± 3.7
	BKA6	20.8 ± 3.9	23.8 ± 1.8	16.3 ± 2.1	12.8 ± 3.4

	BKA1	11.4 ± 1.1	13.1 ± 0.8	13.7 ± 1.3	13.3 ± 2.5
	BKA2	9.6 ± 1.1	12.1 ± 0.8	11.4 ± 0.8	12.3 ± 1.3
RT (%MVC)	BKA3	16.3 ± 1.8	12.4 ± 1.2	15.3 ± 1.5	13.8 ± 1.2
	BKA4	7.6 ± 0.9	13.5 ± 1.0	9.8 ± 0.7	11.0 ± 1.0
	BKA5	11.8 ± 1.4	13.0 ± 0.9	17.7 ± 1.4	18.0 ± 1.6
	BKA6	18.2 ± 1.3	15.4 ± 0.9	12.5 ± 1.2	11.3 ± 1.6

	BKA1	11.9 ± 0.5	11.8 ± 0.4	9.0 ± 0.4	9.1 ± 0.3
	BKA2	9.6 ± 0.4	13.0 ± 0.5	9.8 ± 0.4	9.2 ± 0.4
MFR (pps)	BKA3	8.2 ± 0.5	11.3 ± 0.5	7.3 ± 0.3	11.4 ± 0.5
	BKA4	12.3 ± 0.6	11.1 ± 0.6	9.9 ± 0.3	10.1 ± 0.4
	BKA5	7.0 ± 0.3	8.0 ± 0.3	7.6 ± 0.3	7.3 ± 0.3
	BKA6	7.5 ± 0.3	10.6 ± 0.5	7.4 ± 0.3	8.0 ± 0.3

	BKA1	44.0 ± 2.4	51.6 ± 1.9	28.7 ± 1.2	53.8 ± 1.9
	BKA2	40.0 ± 2.4	107.0 ± 4.8	48.7 ± 2.6	41.6 ± 2.1
PPAmp (*μ*V)	BKA3	30.2 ± 1.8	69.7 ± 3.5	33.4 ± 1.5	61.2 ± 3.0
	BKA4	46.5 ± 2.8	71.5 ± 3.4	28.6 ± 0.9	57.9 ± 2.6
	BKA5	22.0 ± 0.9	33.9 ± 1.5	26.7 ± 1.0	29.3 ± 1.4
	BKA6	27.5 ± 1.1	78.7 ± 4.1	32.5 ± 2.1	32.8 ± 2.0
